# Molecular Research on Platelet Activity in Health and Disease

**DOI:** 10.3390/ijms21113804

**Published:** 2020-05-27

**Authors:** Maria Valeria Catani, Isabella Savini, Valeria Gasperi

**Affiliations:** Department of Experimental Medicine, Tor Vergata University of Rome, 00133 Rome, Italy; savini@uniroma2.it

**Keywords:** cardiovascular disease, microfluidic flow chambers, platelet-cancer cross-talk, platelet activation, platelet concentrate transfusion, stress conditions

## Abstract

This editorial summarizes and discusses the themes of eleven articles (five reviews and six original studies) published in the Special Issue “Molecular Research On Platelet Activity in Health and Disease”. They give an international picture of the up-to-date understanding of (i) platelet signalling under physiological and pathological conditions, (ii) novel technologies for monitoring platelet functions and (iii) clinical applications of platelet-based-therapy for management of pathological conditions, not directly related to haemostasis and thrombosis.

Further insights into the regulation of platelet signalling were derived from Makhoul and collaborators, who report a novel feedback inhibition mechanism in human platelet activity. They report a new site of phosphorylation of spleen tyrosine kinase (Syk), occurring on Ser297, that is mediated by protein kinase C (PKC)- and cyclic adenosine monophosphate (cAMP)-dependent pathways [1]. Considering the central role played by Syk in platelets (as well as in other cells), this finding highlights that a better understanding of Syk regulation is essential for development of drug-based therapies capable of modulating its activity in diseased conditions. 

Grundel and collaborators gave further insights into the functional role of the proteasome system in platelets. Key proteins of this pathway, together with proteins of the ubiquitination system, are indeed expressed by platelets, although the exact role of these proteolytic systems is unclear. By using living *E. coli* in vitro and in sepsis patients, the authors demonstrate, for the first time, that platelets play a key role in sepsis, by increasing proteasome activity, as well as by upregulating the proteasome activator PA28 (PSME1) and inducing proteolytic cleavage of proteasome substrates, such as Talin-1. Upregulation of platelet proteasome activity and protein metabolism in response to infection under conditions of sepsis indicate, therefore, that proteasome is dynamic and responds to inflammatory environmental stress conditions [2]. 

The crucial role of platelets in response to environmental stress conditions is also the object of Kraemer and colleague’s study. They show that acute myocardial infarction leads to significant changes in heat-shock proteins (HSP), acting as chaperones and cytoskeleton stabilizers [3], and kindlins, important proteins involved in integrin signalling and cytoskeleton regulation [4]. Indeed, they report a significant increase of HSP27 protein levels and phosphorylation, as well as intracellular translocation from cytoskeleton to membrane-associated protein fractions in human platelets during myocardial infarction, compared to matched controls with non-ischemic chest [3]. They also describe another platelet phenotype associated to myocardial infarction, i.e., significantly decreased kindlin-3 proteolysis, occurring in soluble and cytoskeletal fractions, but not in membrane fractions [4].

Particular attention should be paid to development of novel technologies for monitoring platelet activity, under both physiological and pathological conditions. In this context, a valuable tool for research in hemostasis and thrombosis is represented by microfluidic flow chambers (MFCs), which can mimic healthy and stenotic blood vessels and recreate various physiological and pathological shear stress conditions. These microchannels, therefore, are commonly used to study platelet adhesion over different adhesive proteins and thrombus formation, as well as inhibitory effects of antiplatelet agents. Nonetheless, the existence of several experimental variables prevents a real standardization, so the definition of common protocols is a compelling challenge. In this context, Scavone’s study is particularly interesting and promising, as the authors investigate critical aspects of microfluidic platelet adhesion assays and of common antiplatelet drugs, i.e., aspirin and cangrelor, through a new microfluidic device [5]. This novel MFC allows us to assess that platelet adhesion on collagen-coated surfaces is a shear-dependent process, not affected by blood storage temperature before perfusion and collagen concentration (beyond a value of 10 μg/mL). Importantly, cangrelor does not inhibit platelet accumulation, at any shear rate and concentration of collagen, while aspirin exerts inhibitory effects at low collagen concentrations. These findings demonstrate the need of considering different aspects of thrombus formation before approaching MFC experiments. A further contribution comes from a novel, easy-to-use, accurate and portable impedance aggregometer prototype called “MICELI” (MICrofluidic, ELectrical, Impedance) designed and fabricated by Roka-Moiia and co-workers [6]. MICELI aggregometer shows several advantages when compared with other commercial devices: it decreases footprint, assay complexity, and time to obtain results. These evidences, together with further validations of operational performance, might give the bases for usage of MICELI aggregometer as diagnostic device for monitoring platelet function during pharmacological thrombosis and bleeding management.

Canault and Alessi exhaustively provide an update of structure and pathophysiological role of RasGRP2, the essential regulator of αIIbβ3 integrin activation in platelets. They show how RasGRP2 genetic variants are related to the inherited platelet-type bleeding disorder-18 (BDPLT18), also discussing strategies for diagnosis and management of patients with this congenital bleeding disorder [7]. Barale and Russo [8] illustrate the intersection complexity between several cardiometabolic risks (among them, obesity, dyslipidemia, impaired glucose homeostasis, hypertension and disturbed microhemorrheology) and thrombosis, focusing their attention on the molecular mechanisms through which all components of metabolic syndrome are involved in prothrombotic tendency observed in obese and diabetic patients. In our Review, we discuss the unexpected central role of platelets in cancer, with particular emphasis on molecular mechanisms underlying platelet-cancer cross-talk and on how modulation of platelet count and secretion (i.e., by bioactive molecules and microvesicle-derived miRNAs) might be related to either a protective or a deleterious action in all steps of cancer progression [9].

Another important aspect in platelet research concerns clinical applications of platelet concentrate transfusion, for treating musculoskeletal conditions (such as osteoarthritis, muscle injuries, tendinopathies, and intervertebral disc degeneration). This is the object of the narrative review from Mariani and Pulsatelli, who discuss different types of methodological procedures used to prepare platelet concentrates and how these preparations may differ in composition, depending on the protocol adopted. Clinical application in musculoskeletal medicine, as well as efficacy and main reported controversies, are illustrated here [10]. The fundamental role of platelets and their derivatives in pathophysiological conditions, not strictly related to hemostasis and thrombosis, is further examined by Salamanna and collaborators: starting from experimental evidence of platelet involvement in bone remodeling, their systematic review highlights the positive correlation between platelet size/volume and bone mineralization, as well as improved bone regeneration by using platelet-derived bioactive growth factors and other derivatives, such as platelet concentrates [11].

Altogether, the studies reported in this Special Issue reveal novel aspects of platelet biology and we hope that they will be helpful towards new insights and a research impetus for those who are interested in developing new therapeutic tools for the management of pathological conditions depending on platelet dysfunctions. 
